# Monte Carlo Simulations indicate that Chromati: Nanostructure is accessible by Light Microscopy

**DOI:** 10.1186/1757-5036-3-11

**Published:** 2010-06-10

**Authors:** Philipp M Diesinger, Dieter W Heermann

**Affiliations:** 1 Institut für Theoretische Physik Universität Heidelberg Philosophenweg 19 D-69120 Heidelberg Germany; 2 Interdisziplinäres Zentrum für Wissenschaftliches Rechnen der Universität Heidelberg Germany

## Abstract

A long controversy exists about the structure of chromatin. Theoretically, this structure could be resolved by scattering experiments if one determines the scattering function - or equivalently the pair distribution function - of the nucleosomes. Unfortunately, scattering experiments with live cells are very difficult and limited to only a couple of nucleosomes.

Nevertheless, new techniques like the high-resolution light microscopy supply a new approach to this problem. In this work we determine the radial pair distribution function of chromatin described by our E2A model and find that the dominant peaks which characterize the chromatin structure are very robust in several ways: They can still be identified in the case of chromatin fibers with reasonable linker histone and nucleosome defect rates as well as in the 2D case after a projection like in most high-res light microscopy experiments. This might initiate new experimental approaches like optical microscopy to finally determine the nanostructure of chromatin.

Furthermore, we examine the statistics of random chromatin collisions and compare it with 5C data of a gene desert. We find that only chromatin fibers with histone depletion show a significant amount of contacts on the kbp-scale which play a important role in gene regulation. Therefore, linker histone and nucleosome depletion might not only be chromatin defects but even be necessary to facilitate transcription.

**PACS codes:** 82.35.Pq, 87.16.A-, 87.16.af

## 1 Introduction

In eukaryotic cells nucleosomes are the basic repeat unit of chromatin fibers [1]. They consist of a central histone octamer and a stretch of DNA (≈150 bp) which is wrapped around it. The histone octamer consists of four pairs of core histones (H2A, H2B, H3 and H4) and is known up to atomistic resolution [2,3]. The nucleosomes are connected by DNA strands of approximately 50 bp length and together with these linkers they form the chromatin fiber. The nucleosome provides the first level of compaction and, furthermore, it is important in the regulation of transcription. Several enzymes can change the position of the nucleosome [4] along the DNA.

The histone H1 is involved in the packing of the beads on a string structure of individual nucleosomes into the 30 nm chromatin structure. It keeps in place the in- and outgoing DNA strand and thus stabilizes the nucleosome. H1 depletion can cause dramatic alterations in the chromatin structure [5].

Access to DNA wrapped in a nucleosome is occluded [6] for polymerase, regulatory, repair and recombination complexes, yet nucleosomes also recruit other proteins through interactions with their histone tail domains [7]. Thus, the detailed locations of nucleosomes along the DNA may have important inhibitory or facilitatory roles in regulating gene expression [8,9].

The chromatin structure is still under discussion [1,10-12]. There are several different structural models: zigzag ribbon models [13-17], helical solenoid models [18-20] or simply having no regular structure [11]. A crystal structure of a tetranucleosome has been revealed [13] and used to construct a model for the 30 nm fiber which resembles a zigzag ribbon that twists or supercoils. The chromatin fiber has been investigated by electron cryomicroscopy [14,21], atomic force microscopy [22,23], neutron scattering and scanning transmission electron microscopy [24]. Beyond the 30 nm level genome folding is poorly understood.

Recent studies [25] showed that linker histones are not necessary for the formation of the 30 nm fiber although they contribute to its compaction. Chromatin compaction does not only depend on the presence of histone H1 and the salt concentration but also on the nucleosome repeat length (NRL) [26] i.e. the length of the DNA stretch that is wrapped around a nucleosome plus the length of the linker DNA that connects two consecutive nucleosomes. Rhodes et al. showed that only the 197 bp NRL can form a 30 nm higher-order chromatin structure and that it shows a cooperative linker histone-dependent compaction. Chromatin strands with a repeat length of 167 bp display a limited linker histone-dependent compaction, which leads to a topologically different thinner fiber. Widom et al. [26] presented a large amount of measurements on NRLs in a previous work. They found that the NRL distributions show preferential quantization to a set of values related by integral multiples of the helical twist of DNA.

Since DNA sequences differ in their ability to bend sharply [27-29] the ability of the histone octamer to wrap different DNA sequences into nucleosomes is highly dependent on the DNA sequence [30,31]. In-vitro studies show this range of affinities to be 1000-fold or greater [32]. Thus, nucleo-somes have substantial DNA sequence preferences which results in a non-regular arrangement of the nucleosomes along the DNA. Furthermore, nu-cleosomes can dissolve entirely by unwrapping the DNA, leaving naked DNA stretches behind, and later on they can reform again. Thus nucleosomes are in a dynamic equilibrium with the chromatin fiber. These effects lead to an average nucleosome occupation of less than 75%. In [33] the average nucleo-some occupancy was partially determined experimentally and predicted by a probabilistic model. Segal et al. extended their model in 2008 to make a prediction for the entire yeast genome [34] and found an average nucleosome occupancy of 68%.

Transcription, especially its initiation, is a complex process. To start the transcription, specific proteins have to assemble at the promoter, which is the DNA region identifying the beginning of a gene. In most of the eukaryotes only a small part of the total genome is dedicated to encode for protein production, for example approximately two percent in human cells. Binding sequences are quite frequently situated about 100 to 200 base-pairs upstream from the promoter and, hence, denoted as upstream elements. But, in particular in eucaryotic cells, regulatory proteins can bind to referring sequences, for instance enhancers, thousands of base-pairs away from the transcription start and still influence the transcription rate. These long-range interactions between regulatory proteins and the transcription complex are facilitated by bending of the intervening chromatin and thus forming a loop. Competition or synergy between proteins, regulating the transcription of the same gene, constitute the basis for a complex gene-regulation network. The efficiency of a regulatory protein depends on its global concentration in the cell nucleus, but also on its local concentration in proximity of the promoter according to the probability of intervening chromatin loops. So, the positioning of a regulatory sequence with respect to the promoter is an important factor, determining the impact of the referring enhancer or repressor protein.

The shape of the genome is thought to play an important part in the coordination of transcription and other DNA-metabolic processes. Chromosome conformation capture (3C) technology allows to analyze the folding of chromatin in the native cellular state at a resolution beyond that provided by current microscopy techniques, although it brings in some other difficulties [35]. 3C technology has become a standard research tool for studying the relationship between nuclear organization and transcription in the native cellular state.

The technique allows the identification of physical interactions between distant DNA segments and of chromatin loops that are formed as a consequence of these interactions, for example between transcriptional regulatory elements and distant target genes [36-40].

Other technologies based on the 3C principle have been developed that aim to increase the throughput: 4C technology allows for an unbiased genome-wide screen for interactions with a locus of choice, whereas 5C technology permits parallel analysis of interactions between many selected DNA fragments. Furthermore, Chip-loop methodology combines 3C with chromatin immunoprecipitation to analyze interactions between specific protein-bound DNA sequences.

It is very hard to analyze chromatin nanostructure by light optical techniques because conventional light microscopy is limited physically to a resolution of about 200 nm, the so-called Abbe limit. Structures below this length scale cannot be resolved by conventional microscopes. Chromatin structures above the level of a single nucleosome, however, are typically in the size range between 10 nm and 800 nm. The diameter of the chromatin fiber lies between 10 nm and 30 nm and renders it impossible to follow the path of the chromatin fiber by conventional light optical techniques. A higher resolution can be gained by using confocal laser scanning fluorescence 4Pi microscopy [41], where laser light is focussed from different sides, allowing for an axial resolution of about 75 nm.

The investigation of chromatin nanostructure, i.e. structure below 100 nm, still faces severe experimental problems. Electron microscopy has been applied to study isolated chromatin segments *in vitro *and thin sections of chromatin *in situ *[18,42,43]. Generally, transmission EM requires a high vacuum and thin samples to allow the beam to penetrate the probe. One way of achieving this is to dehydrate the specimen, embed them in a plastic medium, cut thin sections out of it. Before staining the probe with heavy metals, they have to be chemically fixated due to the invasiveness of the staining procedure raising question to what extend the original structure remains conserved. Obviously, 3D structure information and therefore very important conformational properties are lost by fixating a chromatin fiber to a substrate. A less invasive approach is cryo-EM, where whole unfixed nuclei are used to create frozen hydrated cryosections [44,45]. However, this method too needs very thin section of about 50 nm [43], which possibly disrupts the chromatin structure.

In the last years, there have been advances in light optical techniques allowing for a resolution of single fluorescence labeled molecules with a localization accuracy in the range of about 10 nm. Nowadays, localization microscopy [46-49] allows the determination of single fluorophores with a localization accuracy of single histone molecules. The key idea behind localization microscopy is the following: When passing the optical microscopy setup, each point-like fluorophore will be blurred on the screen, the intensity distribution given by a Bessel function. Only if the distance between two fluorophores is larger than the half-width of the first maximum of this airy disk, the points can be separated. This, however, is not true, if the fluorescent spots have different colors. Then, two points can be *optically isolated *by inspecting the color-dependent maxima, allowing the separation of points much closer than given by the Abbe limit. Optical isolation can also be achieved by utilizing any kind of distinct optical signature, for example different blinking frequencies or consecutive emission times [50]. Spectral precision distance microscopy/spectral position determination microscopy (SPDM) [46-49] uses these different optical signatures to localize photons from a point-like source with an accuracy down to 10 nm. Besides SPDM, there are several other high-resolution light microscopy techniques that do not use this process of stochastic switching, for instance STED [51,52].

In this work we use the E2A-model for chromatin to investigate local chromatin structure properties which are in principle accessible experimentally. Furthermore, we examine the chromatin contact statistics i.e. the statistics of random chromatin collisions or chromatin loops. We will show below that depletion effects and a projection of the whole system do not change the structural characteristics in the nucleosome pair distribution function which could be accessible by scattering experiments or high-resolution light microscopy. We find that histone depletion which leads to disturbed chromatin fibers is not a kind of defect. It allows chromatin contacts on the small length scale of some kbp and thus shows functional aspects because the contacts on this small length scale are important to allow for instance promoter and enhancer regions to come close together. Fibers without histone depletion are much too stiff to have these important loops on the small scale. A comparison with 5C data showed that only the fibers with histone depletion match the experimental results qualitatively.

## 2 Methods

We use the extended two-angle model ('E2A-model') [53-55] based on the local nucleosome geometry and parameter distributions extracted from experimental data to simulate equilibrated chromatin fiber conformations. Our model was extensively described in previous publications [53-55] .

The basis of our chromatin model is the *two-angle model *for chromatin which was introduced by Woodcock et al. [15] to describe the geometry of the 30-nm chromatin fiber. A basic analytical description of the two-angle model including an explanation of the excluded-volume phase transition in the chromatin phase diagram can be found in [53]. We extended the basic two-angle model by introducing a parameter for the distance between the in- and outgoing DNA strand (i.e. the "pitch" of the nucleosomal DNA). An analytical description of the *extended *two-angle model (E2A-model) can be found in [54]. Furthermore, the E2A-model takes into account the excluded volume of the nucleosomes and the DNA as well as the H1 histones that fix the DNA stretches in front of the nucleosomes. The nucleosome repeat length, i.e. the length of the DNA wrapped around a histone plus the DNA linker length is set to 196 bp in this model.

We apply a Monte-Carlo procedure using the model to generate *equilibrated *conformations of chromatin fibers. We generate one chromatin fiber conformation at a time so that we are limited to investigating chromatin in the dilute regime. The fibers in the cell nucleus are of course constrained: They feel the presence of other chromatin fibers and the spatial constraint of the nuclear membrane. Nevertheless, the simulation results can be interpreted as a mean field approximation to the actual fiber conformations since we are only interested in small pieces of chromatin (up to the Mbp scale) and do not try to simulate the whole genome at a time. In this sense our Monte Carlo approach supplies a first order approximation of the actual chromatin fibers.

Simulating chromatin is still a very hard task since the actual interaction potentials (for example of the nucleosome-nucleosome interactions) are hardly known. Our Monte Carlo approach [55] allows us to use probability distributions for the basic model parameters that come from experimental data and thus avoid using interaction potentials (except the well known excluded volume interaction of course). A description of the used probability distributions and how they were derived from experimental data can be found in [55].

**Figure 1:**
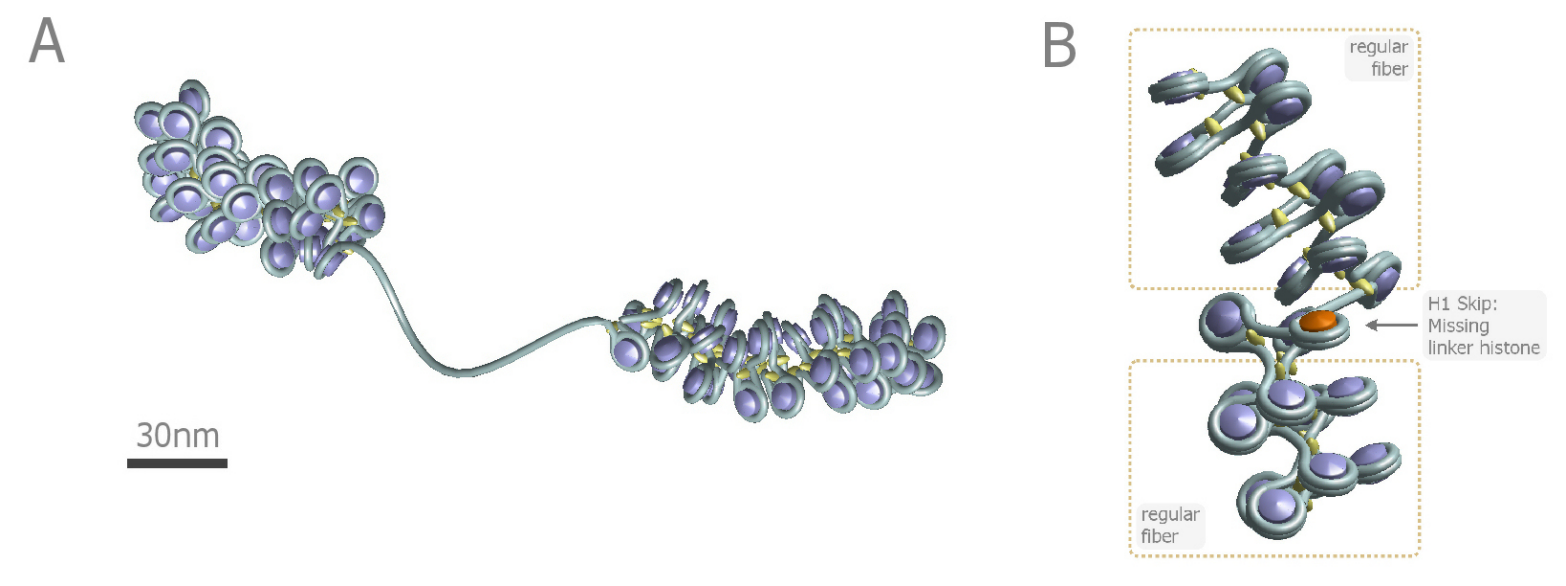
**Illustration of two different histone depletion effects**. A) An example of a single nucleosome skip. If a nucleosome is dissolved, a blank stretch of DNA will remain. The naked DNA stretches have lengths of multiple integers of the nucleosome repeat length plus the length of a DNA linker and can either lead to a collapse or to a swelling of the chromatin fiber [55]. In both cases they increase the flexibility of the chromatin chain massively. B) An example conformation of a short chromatin fiber with a missing linker histone. The upper strand and the strand below the defect are regular, i.e. the local fiber parameters are fixed. Please note that the fiber is very open to make the visualization clear.

**Figure 2:**
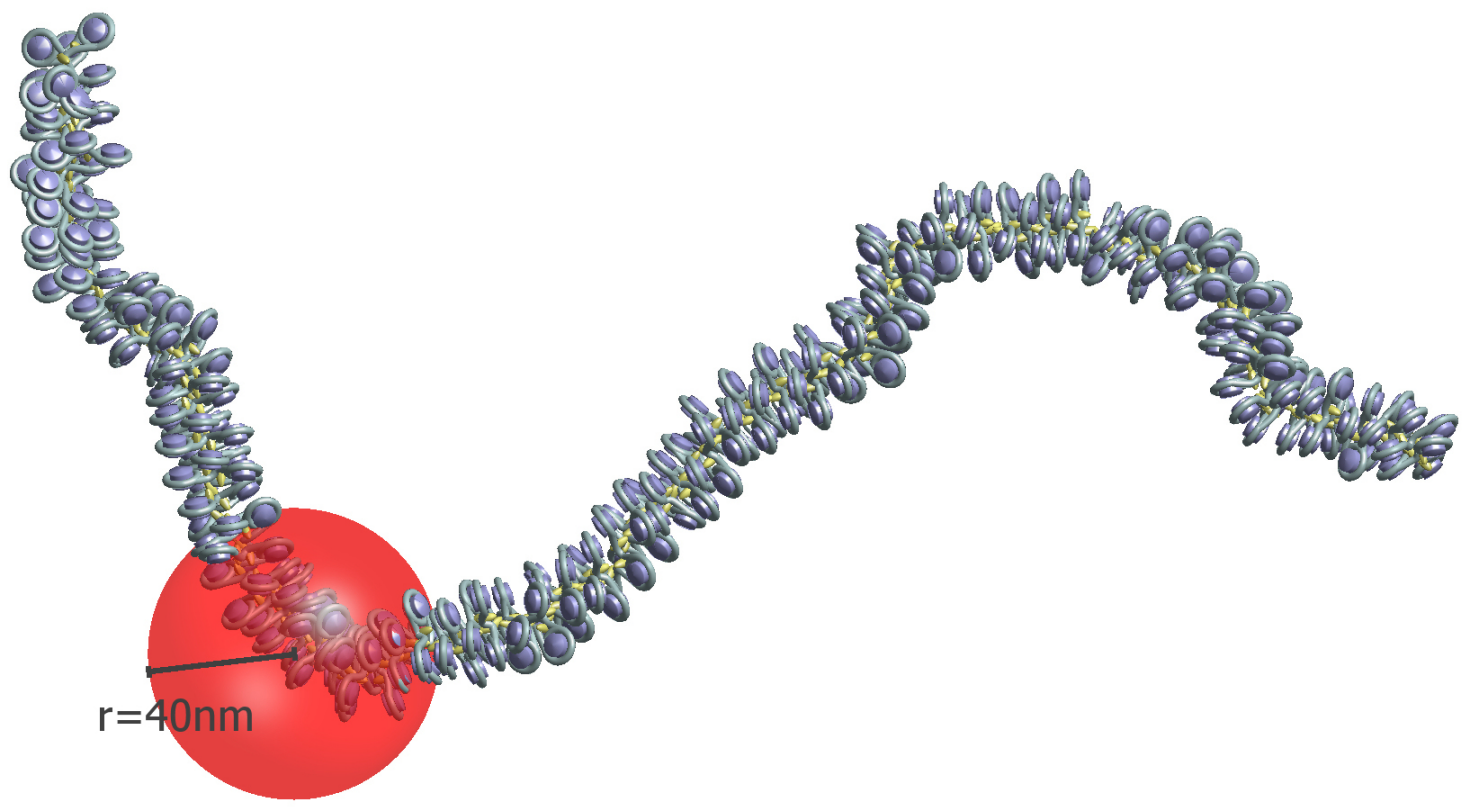
**Illustration of the system size *V *for the structure analysis with the conditional probability *p*(*r*) and the pair distribution function**. The red sphere has a radius of 40 nm

**Figure 3:**
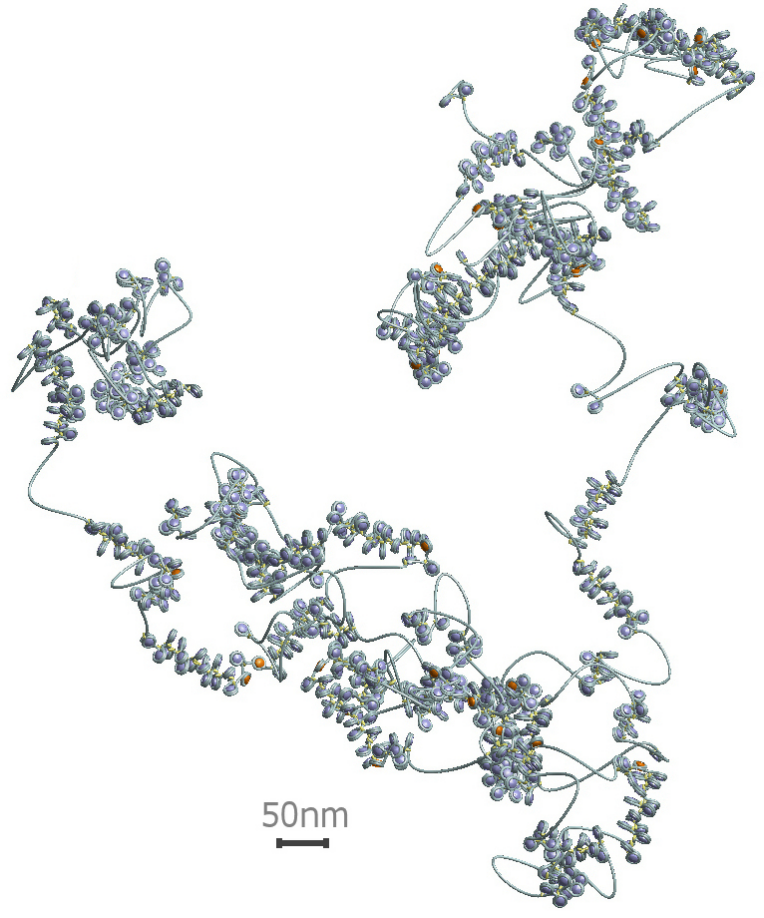
**Example conformation of a chromatin fiber with depletion effects**. The linker histone skip rate is 6% and the nucleosome skip rate is 8%. The linker histone skips are marked orange. One can see that the concept of a regular 30 nm fiber does not hold anymore, if one includes histone depletion. Instead, one gets very flexible coil-like structures of compact regions which are separated by blank DNA stretches. The fiber has a total length of 394 kbp.

The E2A-model can take two different histone depletion effects [55] into account (cf. Fig. 1): *Linker histone depletion*, i.e., missing linker histones which normally would fix the in- and outgoing DNA strand in front of the nucleosome and *nucleosome depletion*. In the latter case not only the linker histone but also the whole nucleosome core particle i.e. the histone octamer is missing so that a long stretch of naked DNA remains. Linker histone depletion gives the chromatin fiber more local flexibility. These two cases have been characterized and discussed in [55]. Fig. 2 shows a part of chromatin fiber without depletion effects whereas Fig. 3 displays a chromatin strand with histone depletion. One can see that as soon as histone depletion is involved the chromatin fiber does not resemble a 30-nm strand any more but gets much more coiled instead. Including the probability distributions for the model parameters also leads from one fixed chromatin structure to a distribution of structures in the chromatin phase diagram that appear with different probabilities. This was discussed in [55].

In this work we used the E2A-model to examine local chromatin structure properties which are in principle accessible experimentally as well as the chromatin contact statistics i.e. the statistics of random chromatin collisions which lead to the formation of loops. If two parts of a chromatin fiber come closer to each other than a previously defined interaction radius r_max _then the part in between is called a 'loop'. In the E2A-model this interaction radius was set to 35 nm.

Furthermore, we investigated if and how depletion effects and a projection of the whole system change the structural characteristics to see whether the chromatin structure might still be characterized under these conditions by light microscopy. Our main metric to describe the chromatin structure is the nucleosome pair distribution function which is accessible by scattering experiments or high-resolution light microscopy.

Chromatin fibers without any depletion effects will be called 'regular' in the following. Fibers with depletion effects will be called 'disturbed'. In the case of the E2A-model the linker histone skip rate was fixed to 6% [54] and the nucleosome skip rate was fixed to 8% [34,55]. In a former publication [55] these skip rates have been treated as parameters to characterize how they change chromatin properties. We found that increasing either one of these skip rates increases the flexibility of the fiber and thus decreases the fiber extension. The persistence length which characterizes the fiber stiffness will decrease from 280 nm to 140 nm if one increases the skip rates from 0% to 8% respectively 6%. If one increases the number of nucleosome skips beyond 8% the fiber starts to swell again because one gets many stretches of blank DNA that occupy space and thus increase the fiber extension again [55]. The same will probably happen if one massively increases the linker histone skip rate (by at least one order of magnitude). The histone depletion skip rates massively affect the fiber's ability to form loops [55].

The simulated lengths of the chromatin fibers in the E2A-model reach from 160 kbp to 1.6 Mbs. We generated at least 10^4 ^fibers for each length. In this model the nucleosomes pack orthogonal to the fiber axis [53-55] . There are also models which consider parallel packing of nucleosomes with respect to the fiber axis [56] but du to that in some cases they have to neglect the DNA trajectories.

## 3 Results and discussion

**Figure 4:**
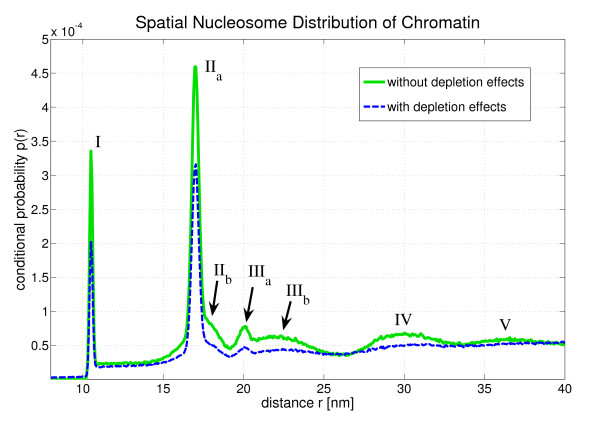
***p*(*r*) is the conditional probability of finding a nucleosome at a spatial distance *r*, if another nucleosome is located at the origin**. One can see a peak structure which comes from the local order of the nucleosomes in the chromatin fiber. The peaks *r*_Δ _can be associated with certain genomic nucleosome-nucleosome distances Δ (cf. Fig. 4). Here it was averaged over approximately 10^14 ^inter-nucleosomal distances.

Fig. 4 shows the conditional probability *p*(*r*) to find a nucleosome at a distance r, if another nucleosome is located at the origin:(1)

where *N *is the total number of nucleosomes and *r*_*i,j*_ denotes the distance of nucleosome *i *and *j*.

We consider two different cases: fibers with and without histone depletion. For the chromatin fibers with histone depletion average depletion rates of 8% (for nucleosome skips) and 6% (for linker histone skips) were applied. These skip rates have been determined experimentally [34,55]. Averages were taken over 10^4 ^chromatin fibers of length 160 kbp, i.e. over approximately 10^14 ^nucleosome-nucleosome distances in total.

**Figure 5:**
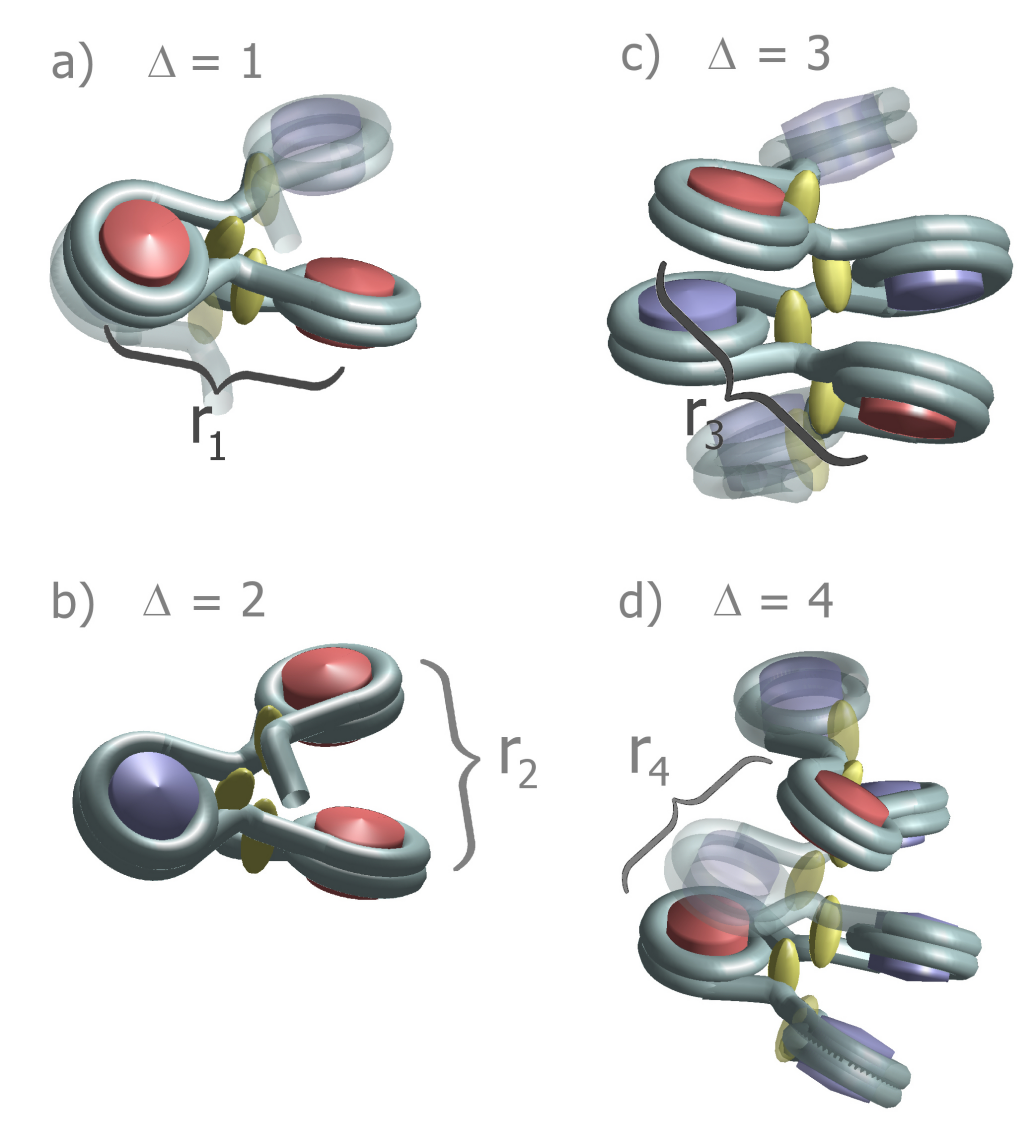
**This figure illustrates the connection between the genomic distance Δ and the spatial distance *r*_Δ _for the first four cases**. The reference nucleosomes which have the genomic distance Δ (given in NRLs) are marked red.

Fig. 4*p*(*r*) shows some very dominant peaks which are labelled I - V. They express the local nucleosome order in the chromatin fiber since they represent very frequent spatial nucleosome distances. They can be associated with certain genomic nucleosome-nucleosome distances. These genomic distances are given in multiples of the nucleosome repeat length, i.e. they are integer numbers. The corresponding spatial nucleosome-nucleosome distance to a genomic distance Δ is denoted by *r*_Δ_. This dependency is illustrated in Fig. 5.

The *r *= 0 peak is not shown in Fig. 4. It has approximately the same height as peak II. *p*(*r*) is almost zero until *r *is larger than 10 nm which is approximately the diameter of the reference nucleosome.

Some of the peaks in Fig. 4 are superpositions of several single *r*_Δ_-distributions. This is indicated in some cases by subscript letters (cf. peak II and peak III). The allocation of the peaks to genomic nucleosome-nucleosome distances can be found in Tab. 1. For instance, the second peak (II) is a superposition of the distribution of *r*_1 _and the distribution of *r*_3_. It is very interesting that the first five peaks of *p*(*r*) can even be identified, if one allows for depletion effects, although in this case *p*(*r*) is decreased in comparison to the conditional probability without depletion effects. This is because linker histone and nucleosome depletion destroy the local order at some points within the chromatin strand so that these spots do not contribute to the dominant peaks any longer.

**Table 1:** Peak Allocation of the Nucleosome Pair Distribution Function

peak #	I	IIa	IIb	IIIa	IIIb	IV	V
genomic distance Δ[bp]	394	197	591	788	985	1182-1379	1576-1970

genomic distance Δ[NRL]	2	1	3	4	5	6,7	8,9,10

Allocation of peak number to particular genomic nucleosome-nucleosome distances for the first five peaks in *p*(*r*) and the pair distribution function *g*(*r*). The peak locations can be found in Tab. 2: They are determined by the most frequent values (mf_Δ_) of the *r*_Δ_-distributions.

Furthermore, the distance distribution with depletion effects is a bit shorter (cf. [Additional File 1]) since the fibers are more flexible [55]. Nevertheless, the first peaks can still be clearly identified.

**Figure 6:**
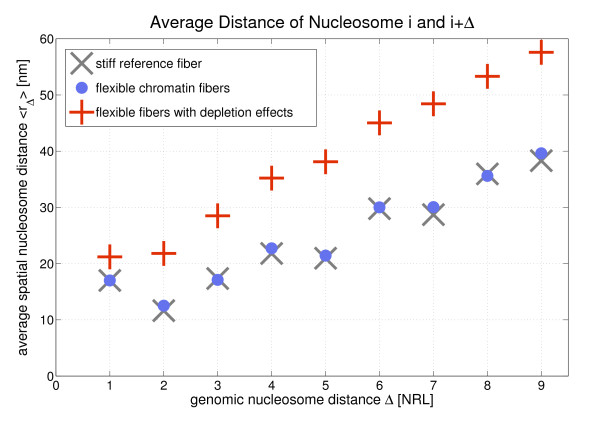
**The mean spatial nucleosome distance 〈*r*_Δ_〉 as a function of the genomic distance Δ for stiff, flexible and flexible fibers with histone depletion**. Some of the *r*_Δ_-distributions are very asymmetric which shifts the mean value far away from the reference value of the regular fiber

Fig. 6 shows the dependence of the average spatial nucleosome distance on the genomic nucleosome distance for three different cases: Stiff fibers, flexible fibers and flexible fibers with depletion effects. One can see that depletion effects strongly *r*_Δ_. This is because depletion effects partly destroy the local nucleosome order. The average of *r*_Δ _was also taken over possibly existing nucleosome or linker histone skips Furthermore, the flexibility and the depletion effects shift the mean values of *r*_Δ _because their probability distributions are very asymmetric in some cases. This can be seen in Tab. 2: The mean value of *r*_Δ _and the most frequent value which is responsible for the peak location differ strongly in some cases.

**Table 2:** Classification of the *r*_Δ_-Distributions for the Chromatin Nanostructure II

	regular fibers			disturbed chromatin fibers		
**Δ**	**〈*r*_Δ_〉**	**σ_Δ_**	**Mf_Δ_**	**〈*r*_Δ_〉**	**σ_Δ_**	**Mf_Δ_**

1.00	51.00	0.63	50.56	63.61	30.72	136.53

2.00	37.55	7.16	31.32	65.42	55.66	31.21

3.00	51.33	4.00	52.37	85.53	62.28	55.42

4.00	68.14	7.17	70.02	105.65	66.40	59.08

5.00	64.27	8.27	66.29	114.37	75.52	7.58

6.00	90.04	6.08	89.94	135.13	74.94	84.41

7.00	90.17	11.13	82.77	145.28	80.88	92.27

8.00	106.81	7.94	58.82	159.95	82.53	3.34

9.00	118.87	10.88	112.72	172.76	85.04	8.36

10.00	125.06	11.55	128.19	183.47	88.61	122.00

This table shows the mean value (〈*r*_Δ_〉), the standard deviation (σ_Δ_) and the most frequent value (mf_Δ_) of the first ten *r*_Δ_-distributions. The first three columns display the values for regular chromatin fibers and the last three columns show the results for disturbed chromatin fibers i.e. fibers with depletion effects. Some of the *r*_Δ_-distribution are very asymmetric since the mean value differs greatly from the most frequent value (e.g. the distribution of *r*_2_). The spatial distances are given in bp and the genomic distances are given in multiples of nucleosome repeat lengths.

**Figure 7:**
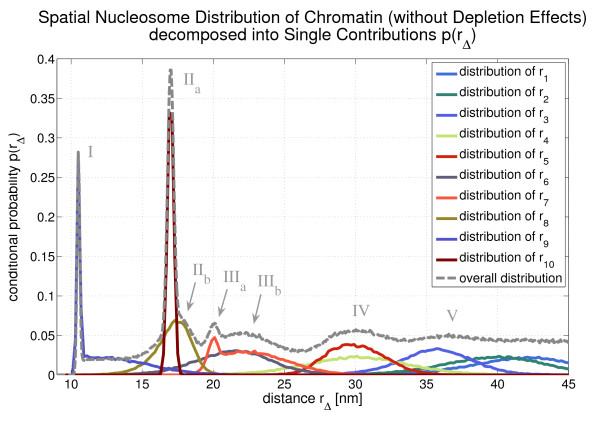
**The overall probability distribution *p*(*r*) is a superposition of the single *r*_Δ_-distributions which is illustrated here for the case of flexible chromatin fibers**. One can see that some distributions are very asymmetric (e.g. *r*_1 _and *r*_7_)

**Figure 8:**
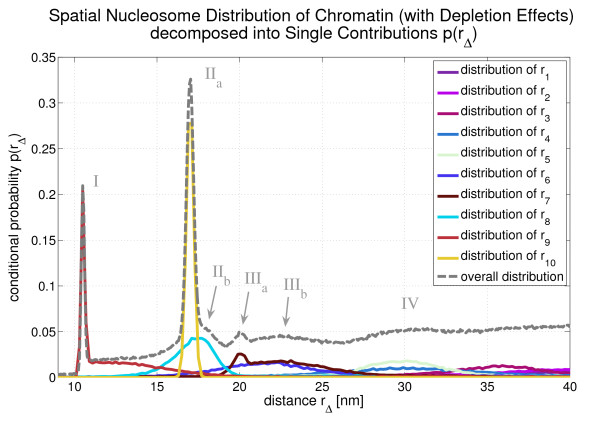
**This figure shows how the different *r*_Δ_-distributions contribute to the overall distribution *p*(*r*) in the case of flexible chromatin fibers with histone depletion**.

The conditional probability *p*(*r*) is the superposition of all *r*_Δ_-distributions. This is illustrated in Fig. 7 for the flexible fibers and in Fig. 8 for the flexible fibers with depletion effects. With the help of these two figures one can determine which peak corresponds to which *r*_Δ_-distribution (cf. Tab. 1). With increasing Δ the distributions of *r*_Δ _become broader and finally the superposition of these distributions, i.e. *p*(*r*) does no longer show a peak structure. This can be seen in [Additional File 1].

**Figure 9:**
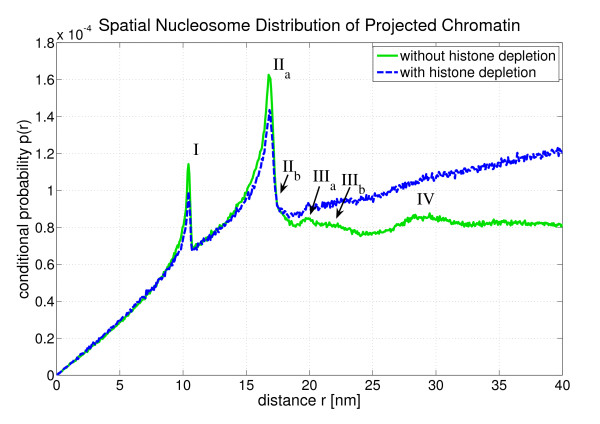
**The radial nucleosome distance distribution in the case of 2D chromatin fibers**. Some of the peaks can still be identified in the case of fibers without depletion effects. Furthermore, *p*(*r*) does not vanish any more for distances smaller than 10 nm. This is an artifact of the projections.

The pair distribution function *g*(*r*) is a major descriptor for the atomic structure of solids, amorphic materials and liquids. Here one can apply this mathematical tool only for small distances because one does not have a chromatin melt but instead only a *single *fiber at a time. Therefore, the distance cut-off for the following structure analysis was set to a small value, namely 40 nm. Thus the spheres with this radius around each nucleosome are analyzed by looking for very frequent spatial distances. This was illustrated in Fig. 2. In this context it is important to keep in mind that the nucleosomes sit at the edge of the chromatin fiber, and furthermore, the fiber itself has only a diameter of about 35 nm. Therefore, the main part of the 40 nm sphere is empty which leads to a decrease of the mean nucleosome density (cf. Fig. 9).

If one nucleosome is sitting at the origin of the coordinate system, the pair distribution function will be proportional to the conditional probability *p*(*r*) of finding another nucleosome at a distance *r*. The probability is normalized such that that a value of one corresponds to the mean nucleosome density of the considered system (i.e. in this case of a 40 nm sphere):(2)

The pair *correlation *function is given by *g*(**r**) — 1 and the Fourier transform of it is the scattering function *S*(**q**) which could in principle be determined by scattering experiments:(3)

In the case of an isotropic system, as we considered here, the scattering function is also isotropic (*S*(**q**) = *S*(*q*)) and one gets(4)

Fig. 9 shows the conditional probability *p*(*r*) Again, the two cases of chromatin fibers with and without histone depletion are considered. There are distances below 10 nm now. These distances do not occur in the 3D chromatin structure and are an artefact due to the projection of the fibers: Some formerly larger distances have been shortened by the projection. A comparison with Fig. 4 shows that all peaks are smeared out towards smaller distances by the projections. This is clear, since projecting vectors can only shorten distances but never increases them. Nevertheless, the first four peaks in the distance distribution function can still be identified in the case of fibers without defects. In the other case of fibers with histone depletion only the first two peaks can be clearly identified.

**Figure 10:**
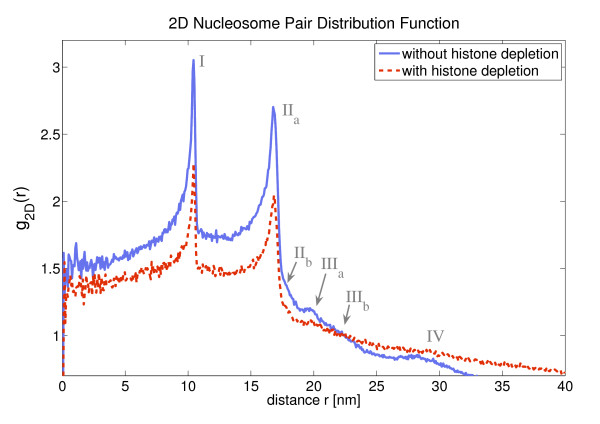
**This figure shows the radial pair distribution function of the nucleosomes in the case of 2D chromatin fibers i.e. projected 3D fibers**. The system size had to be limited to 40 nm since only single model fibers can be evaluated and thus the application of *g*(*r*) is problematic for larger length scales

Fig. 10 shows the two-dimensional radial pair distribution function that corresponds to *p*(*r*) in Fig. 9. For the calculation of the two-dimensional pair distribution function a 2D-sphere with radius 40 nm was used again as the limiting system size. The connection of the conditional probability *p*(*r*) and the two-dimensional pair distribution function is in this case given by:(5)

Δ*r *is the binning parameter and was set to 0.2 bp during the calculations of *g*_2_*D *(*r*) and *R *denotes the 40 nm radius that limits the system size.

2D High-resolution light microscopy is able to produce images with a molecular resolution of the order of some nm. Since there are dyes to label histone molecules within nucleosomes like the H2B histone for instance and the characteristic size of the nucleosomes is about 10 nm it should be possible to access the nucleosome pair distribution function experimentally. Thus, one can establish the local chromatin geometry by the peak structure of the nucleosome pair distribution function even without determining the local nucleosome orientations and also in the case of a two-dimensional projection.

Experimental 3C-based methods [57,58] are very important for the investigation of DNA interactions. We compare the chromatin loop statistics obtained by simulations with the E2A model with actual data sets from 5C experiments [59]. Dekker et al. [59] verified the 5C technology at a previously by 3C experiments investigated region of 400 kbp length around the human *β*-globin locus. Furthermore, they investigated a 100 kb large gene desert on chromosome 16 as a reference system to measure the physical contacts which come from random chromatin collisions and to normalize the data sets obtained by the different experiments. Moreover, two different cell types have been used in this study: The erythroleukemia cell line K562 where the *β*-globin locus is expressed ("On") and the lymphoblastoid cell line GM06990 where the locus is not expressed ("Off"). Since we are only interested in the statistics of purely *random *contacts here we will focus on the part of the 5C data which concerns the 100 kbp gene desert. This region has only the strong interactions between nearby sites but (apparently) no functional long-range looping contacts [57]. Hence, the data should reflect a rather unconstrained chromatin fiber which shows the random coil behavior of chromatin. The *β*-globin region data looks very different from the data of the gene desert and shows some strong long-range looping interactions [59].

**Figure 11:**
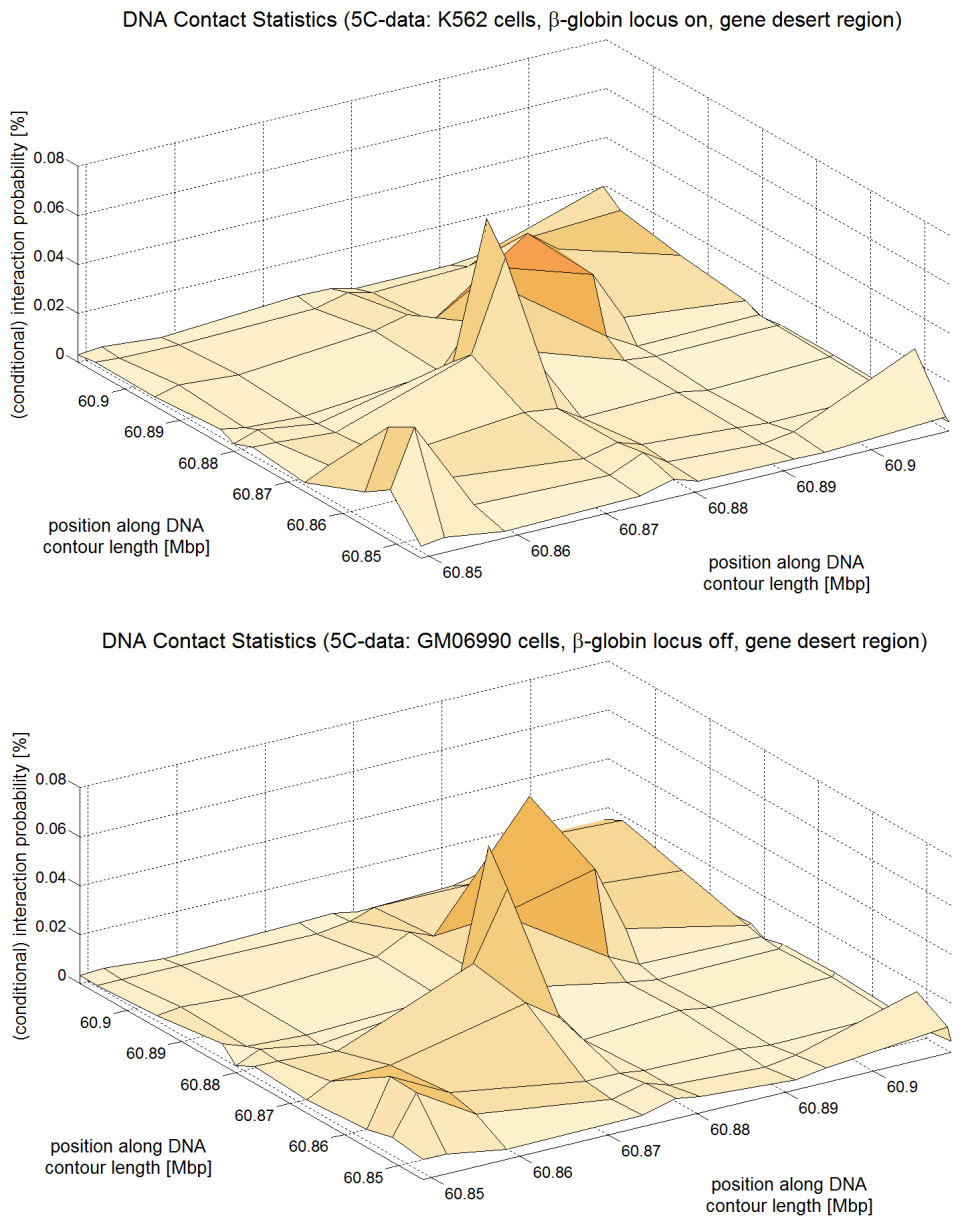
**This figure shows the interaction frequency which was measured by 5C experiments **[59]**on a gene desert at chromosome 16**. The x- and y-axis give the relative position along the genome in Mbp and the z-axis displays their interaction frequency. One can see that chromatin parts that are separated by a small genomic distance interact most frequently whereas chromatin parts that are far apart from each other show hardly any interaction.

**Figure 12:**
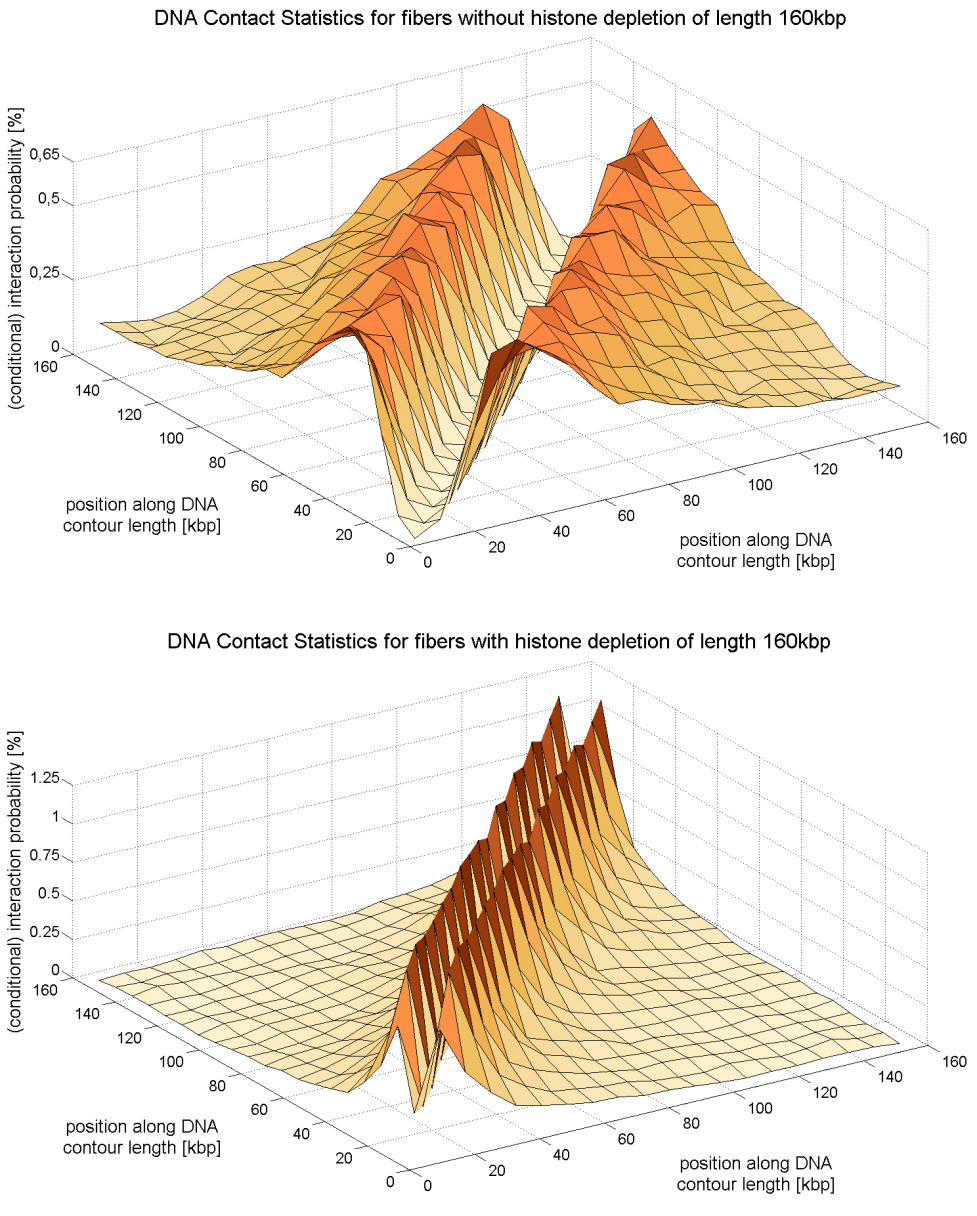
**A similar plot as Fig. 11**. It shows the interaction frequency found by chromatin simulations with and without histone depletion. The fiber length is fixed to 160 kbp in this case. The central gap comes from the local stiffness of the fibers: Chromatin parts without histone depletion which are very close to each other cannot bend towards one another due to the local stiffness of the fiber whereas parts of chromatin fibers with histone depletion can even interact when their genomic distance is small. This mechanism is very important for the genetic activity of the fiber.

**Figure 13:**
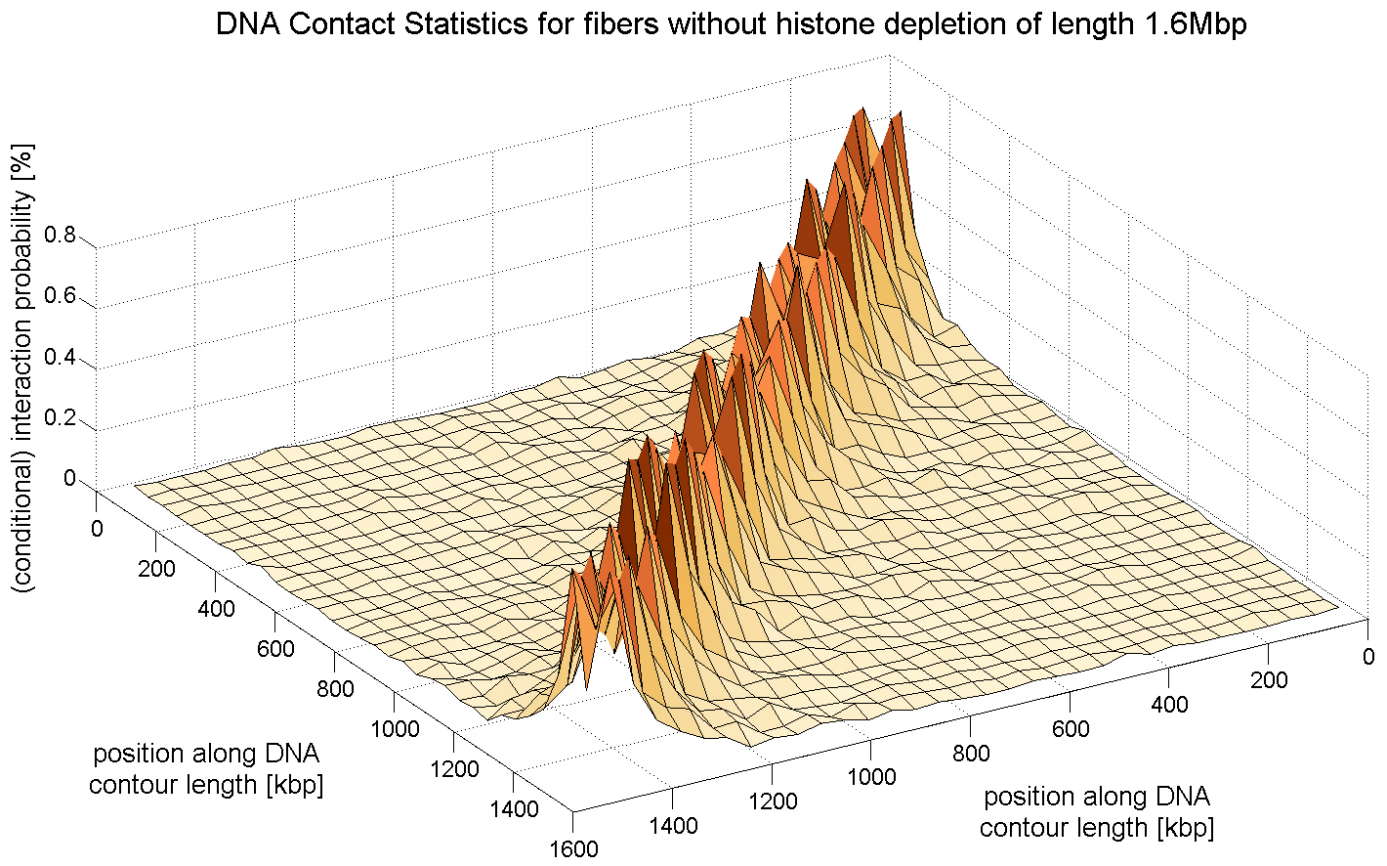
**This figure shows the interaction frequency of much longer chromatin fibers without histone depletion**. The length of the fibers is 1.6 Mbp.

To illustrate the chromatin contact statistics chromatin interaction maps are shown in Fig. 11 (5C-data), Fig. 12 (simulated chromatin fibers of length 160 kbp) and Fig. 13 (simulated fibers without histone depletion of length 1.6 Mbp). One can see that the most frequent interactions occur between chromatin parts that have a small genomic separation.

The large gap in the case of fibers without histone depletion (cf. Fig. 12, top) comes from the local fiber stiffness: Points that are very close to each other along the chromatin fiber can't have a small spatial distance because the fiber is not able to bend that much. The gap occurs in the case of chromatin with histone depletion as well, but its width is much smaller (cf. Fig. 12). The central gaps in Fig. 12 (top) and Fig. 13 are equally broad.

**Figure 14:**
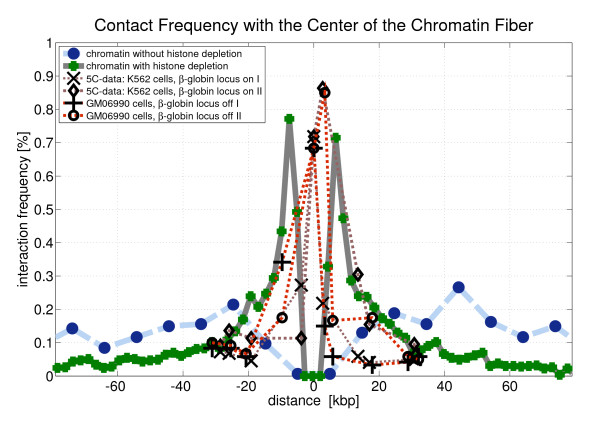
**This figure shows the interaction frequency of different fiber parts with the center of the chromatin fiber in dependence of the distance to the center**. Interactions on the kbp scale are genetically very important for instance to allow enhancers and promotors to come into close proximity to one another. The comparison shows simulation results of chromatin with and without histone depletion as well as results from 5C-experiments of a gene desert on chromosome 16 [59]. The distributions of the simulated chromatin fibers without histone depletion are much to broad to match the experimental data. The fibers would be genetically inactive. The distributions of the simulated fibers with histone depletions show at least a qualitative match of the experimental data. These fibers are genetically active

The gap widths are compared in Fig. 14 where a cut through the previous 3D plots (i.e. Fig. 11, Fig. 12 and Fig. 13) is presented. In the case of the experimental data two cuts have been made (denoted by 'I' and 'II') for each cell type. All cuts go through the central interaction peak and therefore the graphs represent the interaction frequency of a particular fiber part with the center of the fiber. The distance from the fiber center is shown at the x-axis. The sign of the distance corresponds to either one fiber end or the other. One can see that the interaction frequency of chromatin without histone depletion is much too small in order to explain the behavior of the 5C-data. The graph for the fibers with depletion effects comes much closer although some data points seem to lie within the central zero interaction gap. The small chromatin loops i.e. the chromatin contacts on the small scale can only be explained by the fiber with the histone depletion effects. These small loops of loop sizes of about 10 kbp and below are extremely important because they allow promoter and enhancer regions to come close to each other. The fibers without histone depletion do not show this effect because they are far too stiff on this length scale. They have a persistence length of 280 nm which corresponds to a distance along the fiber of 13.5 kbp. Therefore, there are no loops with a smaller loop length than 13.5 kbp in the regular chromatin fibers.

## 4 Conclusions

We investigated the nucleosome pair distribution function of nucleosomes in a chromatin fiber in the framework of the E2A-model and found that it shows some characteristic peaks. These peaks correspond to the local chromatin geometry. We found that they are stable against histone depletion effects i.e. a perturbation of the chromatin fiber. Furthermore, they can even still be identified in the case of a projection of the whole system. Since two-dimensional high-resolution light microscopy can resolve single molecules it is in principle possible to determine the nucleosome pair distribution function experimentally and thus establish the local chromatin geometry.

We find that histone depletion which leads to disturbed chromatin fibers is not a kind of defect. It allows chromatin contacts on the length scale of some kbp and thus shows functional aspects because the contacts on this small length scale are important for instance to allow promoter and enhancer regions to come close together. Fibers without histone depletion are much too stiff to have these important loops on the small scale. A comparison with 5C data showed that only the fibers with histone depletion match the experimental results qualitatively.

## Supplementary Material

Additional file 1 **Supplementary Figure**. This figure displays the previously discussed conditional probability *p*(*r*) on the large length scale. Click here for file
