# Supplementary figures and images for: Monte Carlo Simulations indicate that Chromati: Nanostructure is accessible by Light Microscopy
Source: PMC Biophys. 2010 Jun 10;3:11. doi: 10.1186/1757-5036-3-11 (PMC2911407; doi:10.1186/1757-5036-3-11)

# Spatial Nucleosome Distribution of Chromatin

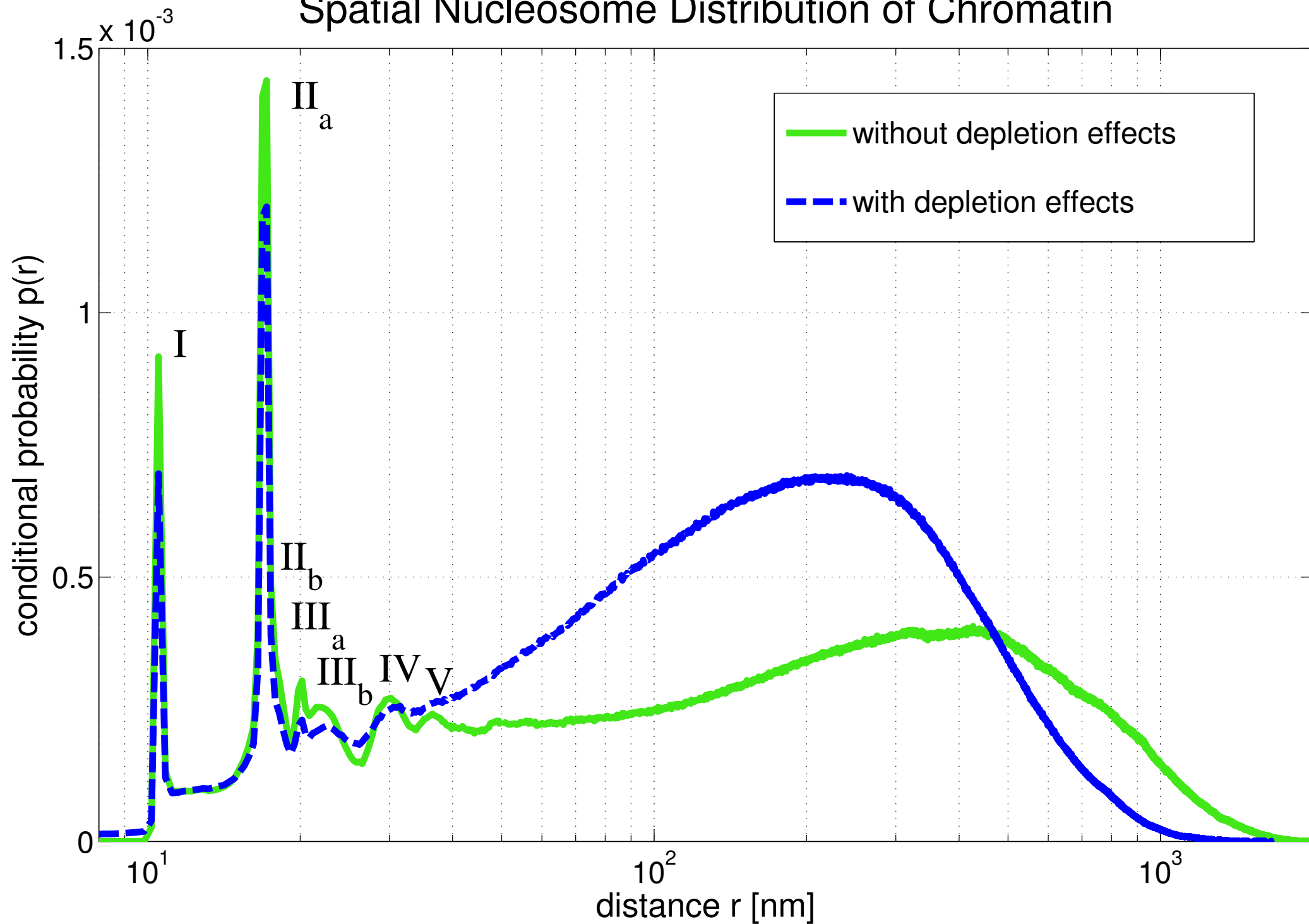

Supplement: Additional file 1 — Supplementary Figure. This figure displays the previously discussed conditional probability p(r) on the large length scale. [file 1757-5036-3-11-S1.PDF]
